# Editorial: Cardiovascular diseases in autoimmune diseases: Dyslipidemia and vascular inflammation

**DOI:** 10.3389/fcvm.2022.1043669

**Published:** 2022-11-22

**Authors:** E. Blair Solow, Chieko Mineo

**Affiliations:** ^1^Division of Rheumatic Diseases, Department of Internal Medicine, UT Southwestern Medical Center, Dallas, TX, United States; ^2^Center for Pulmonary and Vascular Biology, Department of Pediatrics, UT Southwestern Medical Center, Dallas, TX, United States

**Keywords:** rheumatoid arthritis, systemic lupus erythematosus, antiphospholipid syndrome, atherosclerosis, dyslipidemia, lipoprotein, cardiovascular disease

Chronic inflammatory autoimmune disorders such as rheumatoid arthritis (RA), systemic lupus erythematosus (SLE) and anti-phospholipid syndrome (APS) are life-threatening disorders that affect millions of people worldwide. These conditions have long been associated with an increased risk of cardiovascular diseases (CVD), such as myocardial infarction (MI) and ischemic stroke (1–4). Premature cardiovascular mortality drives substantially shortened life expectancies in the patients with the autoimmune disorders. While traditional risk factors associated with CVD including smoking, dyslipidemia, diabetes mellitus, hypertension and obesity are present in RA and SLE patients, standard Framingham scores do not fully explain the high rates of CVD events. Thus, autoimmune diseases are regarded as an independent risk factor for the development of cardiovascular comorbidity. Dyslipidemia is highly associated with CVD in general population, and statin therapies targeting lower LDL-cholesterol levels are an effective intervention for prevention of CVD. Interestingly, abnormal lipid profiles are also observed in RA or SLE patients (5). In addition to dyslipidemia, chronic inflammation has been implicated as a key contributor to atherosclerosis, a major underlying cause for CVD (6). However, it remains unclear whether dyslipidemia or inflammatory variables can serve as biomarkers to predict future CVD events or whether they play a causal role in CVD development in patients with the autoimmune disorders (7, 8).

This Research Topic aims to advance the understanding of the role of inflammation and dyslipidemia in CVD-related pathogenesis in the inflammatory autoimmune disorders RA, SLE, and APS, hoping to provide a perspective that may lead to development of novel therapeutic interventions and more effective preventive modalities.

In these studies presented in the Topic, cardiovascular disease was assessed at macro levels by investigating imaging techniques including pulse wave velocity to assess the arterial distensibility and by evaluation of comorbid conditions and lab testing associated with CVD events. In turn, looking more deeply at the micro level at biomarkers that could play a predictive role in developing CVD which could change the course of the disease. At the macro level, it was investigated if an inflammatory condition such as RA could drive changes in vascular imaging that is associated with CVD. Patients with RA are at considerable risk for CVD (3), and aging is one of the major contributors for CVD in both general and RA populations. The work by Rodríguez-Vargas et al. assessed the impact of chronic, low-grade inflammation (termed inflammaging) and vascular dysfunction associated with aging in RA. This cross-sectional study made a comparison in vascular age between RA patients with low disease activity due to a strict treat-to-target strategy (*n* = 52) and those with osteoarthritis (OA) with poor metabolic control (*n* = 54). The results found no differences between the two groups in vascular age, as measured by pulse wave velocity (PWV), and additional inflammaging markers. The finding suggests that despite aggressive control of the disease, RA patients have similar vascular dysfunction to OA patients with the metabolic syndrome. Although there are limitations of the study that include the need for a healthy control comparator, uncertainty regarding sensitivity and efficacy of PWV as an indicator of vascular age in RA population, and the cross-sectional nature of the study (9), this work demonstrates that vascular age, possibly accelerated by chronic inflammation, can be a helpful tool for CVD risk stratification of RA patients.

If inflammation can affect the vasculature negatively, can it be prevented or reversed? In addition to traditional risk factors, such as age, sex, blood pressure and diabetes, RA itself and RA-associated inflammation are independent risk factors for atherosclerosis. Thus, it is important to understand how therapeutic drugs commonly used for RA influence the development of CVD. The review by Baoqi et al. focuses on how therapeutic drugs for RA, including non-steroidal anti-inflammatory drugs (NSAIDs), glucocorticoids, disease-modifying anti-rheumatic drugs (DMARDs), and botanical products impact cardiovascular events. The study provides a comprehensive list of RA medications and in-depth discussion of potential mechanisms by which each class of medications affects CVD development. Biologic medications blocking TNFα may limit CVD development. The review emphasizes the importance of weighing the advantages and disadvantages of different medications and of planning individualized treatment plans suitable for RA patients.

Thromboembolic events can be fatal if not treated acutely. The following two studies investigate comorbid conditions and routine laboratory data that could help predict the development of disease. APS is strongly associate with increased risk for thrombosis, and it is also associated with recurrent pulmonary embolism (PE). The work by Shi et al. investigated the clinical characteristics of APS patients with PE, aiming to develop a novel risk score system for APS patients presenting with an acute PE. In this single center retrospective study, 76 patients, who developed a PE and tested for antiphospholipid antibodies, were categorized into APS (*n* = 46) and non-APS (*n* = 30) based on the Sydney criteria (10). In the APS group approximately 2/3 were primary APS, 1/3 had SLE, and one patient had recurrent miscarriages. When comparing the APS to non-APS group after controlling for age and sex, the risk factors for APS included male, decreased platelets, increased D-dimer and prolonged partial thromboplastin time, termed MPDA risk score. The authors suggest MPDA score should be taken into account for screening for antiphospholipid antibodies in patients presenting an acute diagnosis of PE of unexplained etiology. However, this is a small cohort at a single center, and additional prospective analyses are needed to confirm the validity of the proposed prediction model.

Previous studies have found an increased risk for MI in APS patients. Gan et al. investigated the clinical characteristics of APS patients who developed acute MI. A retrospective cohort study was performed in 332 patients with APS and 239 patients with thrombotic APS, with 5 year follow-up. Twelve percent of the patients in the study developed MI, and after adjusting for age and gender, the study identified several factors that were positively associated with acute MI in APS patients. These include multiple organ thrombosis, atherosclerosis, and elevated neutrophil count. Interestingly, the venous thrombosis was negatively associated with acute MI. Future studies with larger, prospective cohorts will further determine whether these clinical and laboratory findings in APS associated with MI can be explored as novel predictors or therapeutic options.

Inflammation leads to vascular change and the Baoqi et al. study and many others suggest targeting inflammation can decrease the risk of developing CVD in RA. The next step is understanding mechanistically how these changes occur. Inflammation and dyslipidemia are major contributors of atherosclerotic CVD; however the interaction between inflammation and lipid profiles remains largely unknown. It is well established that RA is associated with disrupted circulating lipid profiles (3). Anti-tumor necrosis factor (TNF) therapies relieve disease activity and they also decrease CVD risk in RA, but their comprehensive effects on the lipid profile have not been well described. The study by Luo et al. investigated the effects of anti-TNF therapies on blood lipid profiles in RA patients. Through meta-analysis of MEDLINE, Embase and the Cochrane Central Register of Controlled Trials (CENTRAL) database, a total of 44 records and 3,935 patients were included in the study. Anti-TNF therapies were associated with significant increase in total cholesterol (TC) and high-density lipoprotein (HDL), regardless of the duration of the treatment; anti-TNF therapies were associated with increased low-density lipoprotein (LDL) and apolipoprotein A1 (ApoA1) in the short-term, but not in the mid-term and long-term; triglyceride (TG) and apolipoprotein B (ApoB) do not change; proatherosclerotic indexes (ratios of TC/HDL, ApoB/ApoA1, and LDL/HDL) tended to decrease in the short- and mid-term, but returned to baseline in the long-term after TNF inhibition. Anti-TNF therapies were related to a long-term elevation in HDL levels, which, together with evidence of improved HDL function, may contribute partially to the decreased CVD risk by TNF inhibition. Combined with the previous meta-analyses (11, 12), the report further confirms the effect of anti-TNF therapies on TC and HDL, and it provides additional information on the short-term vs. long-term effect of the therapy on lipid profiles. The mechanism of how TNFα inhibition results in the observed changes in TC and HDL remains unclear. Future investigations focusing on the pro-inflammatory nature of lipid profiles in CVD development in RA are warranted.

To look further at the micro level in the pathogenesis of CVD, the comprehensive review of low-density lipoprotein receptor-related protein-1 (LRP1) in atherosclerosis by Chen et al. highlights the influence of LRP1 during atherosclerosis development, focusing on its dual role in vascular cells and immune cells. LRP1 belongs to the LDL receptor (LDLR) gene family, which regulates diverse physiological and pathological processes including plasma lipoprotein homeostasis, inflammation and atherosclerosis. LRP1 plays an anti-atherogenic role through removal of lipoproteins in the liver; however, it also facilitates the uptake of aggregated LDL to promote the formation of macrophage- and vascular smooth muscle cell (VSMC)-derived foam cells. The dual and opposing roles of LRP1 may also represent an interesting target for atherosclerosis therapeutics. LRP1 can be cleaved by cell surface proteases to produce soluble LRP1 (sLRP1), and this process can be accelerated by inflammatory mediators in macrophages, such as lipopolysaccharide and interferon-γ. sLRP1 maintains the ligand-binding properties and may act as a competitive inhibitor for a ligand that binds LRP1 on the cell surface. sLRP1 was shown to promote TNF-α, monocyte chemoattractant protein type-1 and IL-10 expression in monocytes. Interestingly, increased levels of circulating sLRP1 have been reported in patients with RA and SLE (13). This review calls attention to the limited available knowledge regarding LRP1 or sLRP1 contribution to CVD in RA, paving the way for future investigations to explore the role of lipoprotein receptors and their signaling in vascular cells (endothelial cells, macrophages and smooth muscle cells) in atherogenesis in RA.

The rise of metabolomic research has further advanced the field in sourcing biomarkers and perhaps shed light on pathogenesis, to predict organ damage, comorbidity such as CVD and other features, such as in SLE. The study by Baig et al. employed a comprehensive metabolomic screening of sera from SLE patients to identify metabolites that predict future carotid plaque progression. Three groups of subjects were studied after 8–9 years of follow-up; SLE patients without plaque progression (*n* = 9), SLE patients who went on to develop atherosclerotic plaques (*n* = 8), and controls without SLE (*N* = 8). In this OMICs study investigating SLE and atherosclerotic carotid plaque, glucogenic and ketogenic amino acids metabolites differentiated SLE who developed plaques from SLE without plaque progression or from non-SLE controls. In the screen, metabolites in the n6-PUFA/arachidonic acid lipoxygenase pathway, such as LTB4, 9-HODE, and 13-HODE, were found to be elevated in SLE sera with carotid plaque. Availability of the longitudinal data and the large number of metabolites for future analyses are strengths of this study, although the small sample size did not allow the authors to address issues of confounding factors such as medications, comorbidities and smoking. The current work provides a strong rationale for future studies to assess whether these metabolites serve as biomarkers to predict the development of CVD in SLE patients.

In summary, autoimmune diseases such as RA, APS, and SLE have aberrant inflammatory pathways and dyslipidemia ultimately leading to CVD (14). These studies presented in the Research Topic provide up-to-date information regarding how inflammation and dyslipidemia in RA, SLE and APS can be reversed to combat devastating CVD comorbidity. We hope the Research Topic further stimulates the research field to move the science forward through multi-faceted macro and micro approaches, including modalities to assess vascular health such as aortic pulse wave velocity, OMICs to identify novel predictive biomarkers, and human cohort studies to assess risk factors and effects of medications on CVD associated with inflammatory autoimmune disorders (15, 16).

## Author contributions

Both authors listed have made a substantial, direct, and intellectual contribution to the work and approved it for publication.

## Funding

This work was supported by the AHA Transformational Project Award (957261) for CM and ES.

## Conflict of interest

The authors declare that the research was conducted in the absence of any commercial or financial relationships that could be construed as a potential conflict of interest.

## Publisher's note

All claims expressed in this article are solely those of the authors and do not necessarily represent those of their affiliated organizations, or those of the publisher, the editors and the reviewers. Any product that may be evaluated in this article, or claim that may be made by its manufacturer, is not guaranteed or endorsed by the publisher.
